# Supplementary figures and images for: In vitro activity of ceftazidime/avibactam, cefiderocol, meropenem/vaborbactam and imipenem/relebactam against clinical strains of the Stenotrophomonas maltophilia complex
Source: PLoS One. 2024 Apr 18;19(4):e0298577. doi: 10.1371/journal.pone.0298577 (PMC11025899; doi:10.1371/journal.pone.0298577)

## Slide 1
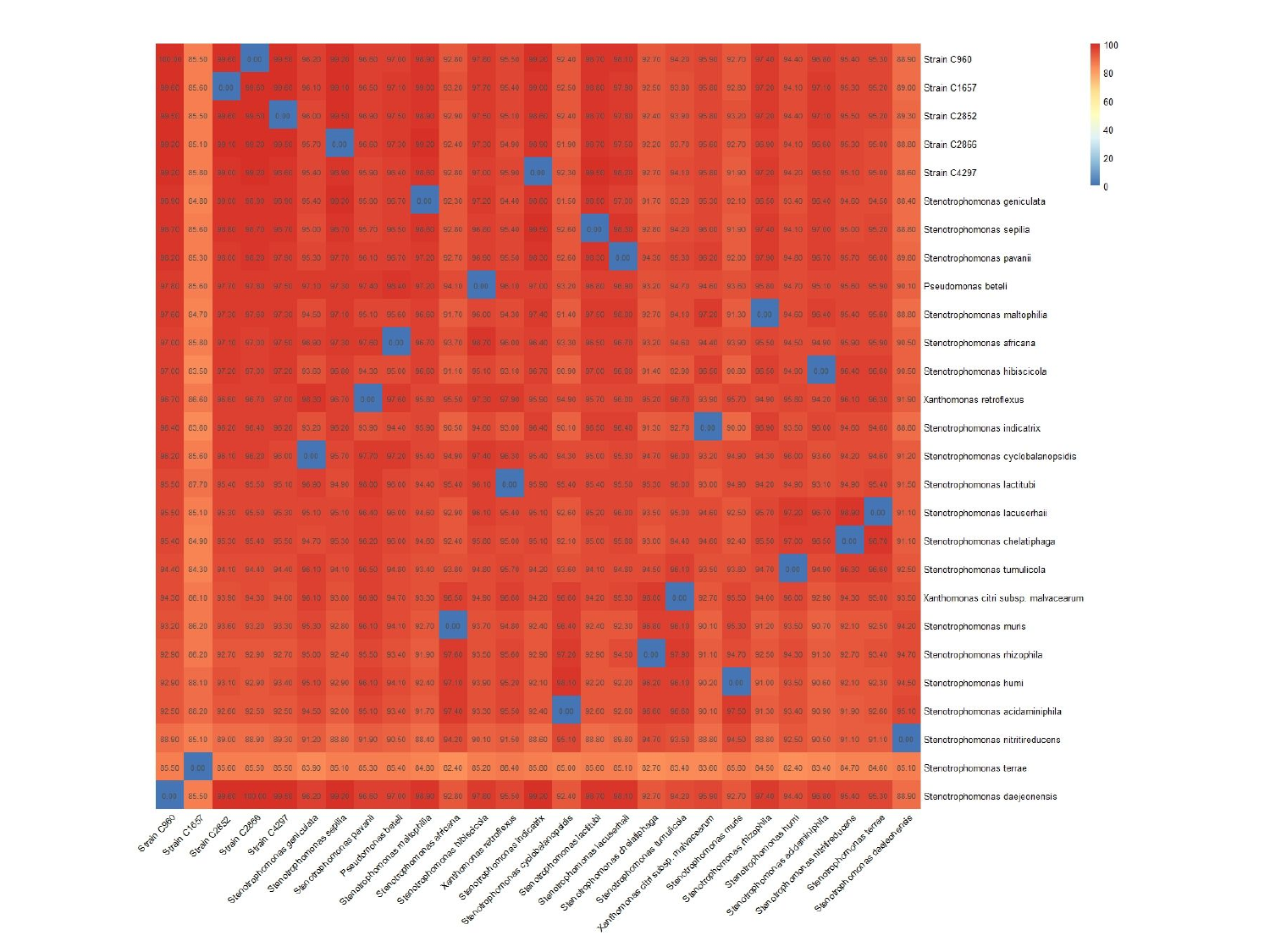

Supplement: S1 Fig — Degraded panel on the right side reflects the visual representation of identity percentage from the lowest value (0% in blue) to the highest value (100% in dark red). Numbers into the boxes represent the numerical value of the identity percentages. (PPTX) [file pone.0298577.s001.pptx]
