# Supplementary material for: In vitro activity of ceftazidime/avibactam, cefiderocol, meropenem/vaborbactam and imipenem/relebactam against clinical strains of the Stenotrophomonas maltophilia complex
Source: PLoS One. 2024 Apr 18;19(4):e0298577. doi: 10.1371/journal.pone.0298577 (PMC11025899; doi:10.1371/journal.pone.0298577)
Supplement: Attachment — Submitted filename: Response to Reviewer.docx [file pone.0298577.s003.docx]

Rebbutal letter

First of all, we would like to thank the reviewers for their time and comments on this paper.

Reviewer 1. L162: Of these, 118 (53.8%) were excluded from the analysis for various reasons. For the authors to clarify the reasons behind the exclusión.

Thank you very much for the observation. We wrote, sic- Of these, 118 (53.8%) were excluded from the analysis for various reasons: two were environmental samples, 32 were swab samples, 14 strains were recovered from catheter tips, one from sonication of prosthetic joint material, four had missing information in electronic medical records, 19 were from urine cultures, and 46 were repeated isolates.- We decided to include only strains related with valuable microbiological samples, since microorganisms isolated from swabs could be colonizers rather than infectious. Same case for environmental samples (from beds, surfaces, etc.), for catheter tips the exclusion reason was because we included those strains isolated from blood cultures (central or peripheric), the case for sonication was similar, we decided to include the strain from biopsy, 4 strains did not have information in the electronical record and we wanted to reduce the selection bias to reduce the overestimation, then those repeated strain from a patient were excluded and we only include one from each patient.

Reviewer 1. Fig 1: I would suggest to remove it as it is not providing additional information

I would rather suggest to use a table with the following data :

Patient Demographic (Age, sex)

Site of isolation

Date of isolation

Patient location ( ex, ICU, CCU)

Thank you very much for the observation and recommendation. At the beginning we though to include the data mentioned above, however, the purpose of the study was centered only in the susceptibility pattern. Since we decided to exclude some strains, the inclusion of these variables is in order to express an epidemiological behavior of our strains. We are interested in further works with *Stenotrophomonas spp.*, that’s why, in this case, we did not include location and demographic data. Nevertheless, as a supplementary table we included strain, date, and site of isolation (Table S1). If you do not have problem, we consider an easy way to follow the number of strains with the algorithm included.

Reviewer 1. For the result part, I would recommend to change the way of presentation ( ex-Table 1 and Table 2 to be merged in one table.

You are right with the observation since both tables are linked, many thanks. The first table is the complete MIC distributions for each antibiotic and second one, which depends on the first, is about the concentrations were the most went down. We tried same to adjust as your recommendation; however, it was difficult to follow due to there were several values (for MICs concentrations). We consider it simpler to avoid getting lost along the reading and not confusing the reader.

Reviewer 1. In Figure 2, the authors presented the trend in sensitivity over the 10 years.
However, it is not clear how many strains have been isolated every year in order to conclude whether those percentage are significant or not

Thank you very much. According to your initial recommendation, in Table S1 are included strains’ isolation dates.

Reviewer 1. L301: We found that we imported two 301 strains from other hospitals
Could the authors elaborate more on this?

Thank you very much for the observation. We added the following sentence sic- because the Instituto Nacional de Rehabilitación Luis Guillermo Ibarra Ibarra is a third level center, some cases came to our institution from the first instance and many other patients were referred from other hospitals. Once the patient arrives, we perform the microbiological protocol to know their colonization status with which the patient arrives. In this way, we may correlate the cases of importation of those residents ones-

Reviewer 1. L409: This positions it as a viable option for comparison with other antibiotics, including 410 monobactams.

It would be a great added value if the authors would be able to test the combination of Ceftazidime avibactam and Aztreonam and share the findings

This is a great observation, and a huge opportunity niche as you suggest. As you are thinking, ATM will be interesting since *S. maltophilia* has a metallo ß-lactamase, unluckily, in this moment we do not have aztreonam salt to perform the experiments, neither more avibactam, we decided to include them, but we did not have enough salt, that´s why we did the observation as limitation of our study. However, for sure, it would be interesting to prove it, in combination, in one side avibactam able to inhibit the cephalosporinase and second, aztreonam evading the action of metallo and then, in combination with ceftazidime. As we say before, we are planning to work with *Stenotrophomonas* and for sure, we will test ATM/ceftazidime/avibactam and we think to include another inhibitor of metallo ß-lactamases. On the other hand, we did not have in that moment ATM in Mexico, and hence there is not ATM available as treatment.

Reviewer 2.

We want to thank you very much for your time taken to review our work.

Thank you very much, we really appreciate it.
